# Evolving and Novel Applications of Artificial Intelligence in Abdominal Imaging

**DOI:** 10.3390/tomography10110133

**Published:** 2024-11-18

**Authors:** Mark R. Loper, Mina S. Makary

**Affiliations:** Department of Radiology, The Ohio State University Wexner Medical Center, Columbus, OH 43210, USA; mark.loper@osumc.edu

**Keywords:** artificial intelligence, abdominal radiology, machine learning, imaging, pancreatic, colorectal, gastric, hepatic

## Abstract

Advancements in artificial intelligence (AI) have significantly transformed the field of abdominal radiology, leading to an improvement in diagnostic and disease management capabilities. This narrative review seeks to evaluate the current standing of AI in abdominal imaging, with a focus on recent literature contributions. This work explores the diagnosis and characterization of hepatobiliary, pancreatic, gastric, colonic, and other pathologies. In addition, the role of AI has been observed to help differentiate renal, adrenal, and splenic disorders. Furthermore, workflow optimization strategies and quantitative imaging techniques used for the measurement and characterization of tissue properties, including radiomics and deep learning, are highlighted. An assessment of how these advancements enable more precise diagnosis, tumor description, and body composition evaluation is presented, which ultimately advances the clinical effectiveness and productivity of radiology. Despite the advancements of AI in abdominal imaging, technical, ethical, and legal challenges persist, and these challenges, as well as opportunities for future development, are highlighted.

## 1. Introduction

Diagnostic imaging continues to undergo rapid change as technology advances, improving the diagnostic capabilities within medical imaging. This impact is particularly evident in abdominal and thoracic imaging, with cutting-edge developments, such as artificial intelligence (AI), helping to identify, diagnose, and manage diseases. A recent comprehensive review paper was published on thoracic applications of AI [1]. Similar review papers focusing on abdominal applications of AI have been published [2,3,4]. However, given the rapid rate at which new AI applications are emerging in radiology, this narrative review highlights many of the latest AI applications, focusing on novel developments within the past few years and selectively incorporating relevant advancements from earlier years to provide historical context. A primary focus is to summarize findings from manuscripts in which more than 300 patients/images were studied using AI intervention. However, smaller studies are included when deemed relevant for context in the topic being studied. 

**Figure 1:**
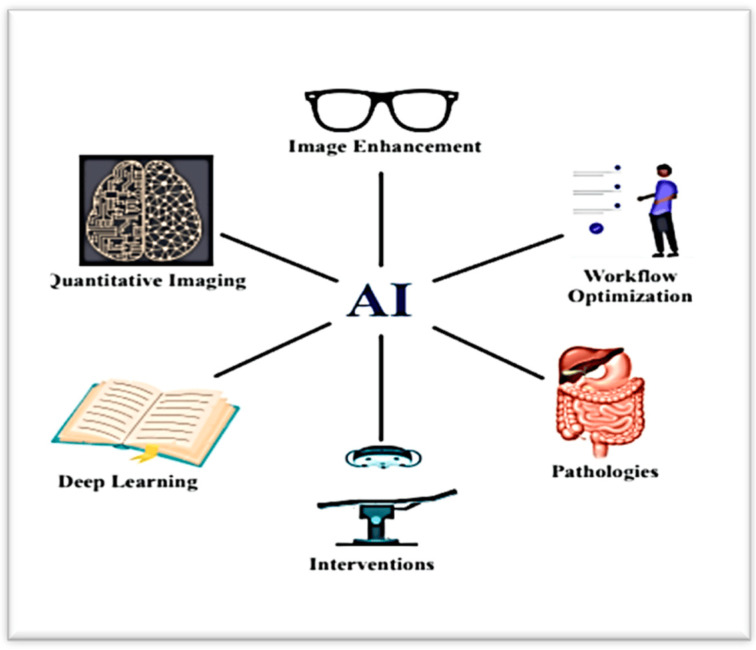
Summary of AI applications in abdominal radiology.

This narrative review provides a focused discussion of AI applications in abdominal radiology (Figure 1). Specific abdominal pathologies by organ system are discussed, which includes hepatic, pancreatic, gastric, colorectal, and others, offering a body-part-specific perspective on AI’s role in abdominal radiology. Each section addresses the valuable information AI can provide to assist with the diagnosis, grading, and prognosis of these diseases. Moreover, advanced image processing and analysis play a role in interpreting abdominal imaging, and those topics are covered here. Image enhancements using reconstructive AI algorithms and quantitative imaging techniques are discussed as well. Beyond diagnostics, this narrative review discusses how AI optimizes workflow within radiology and assists in minimally invasive procedures. Although abdominal imaging has been transformed in many ways by AI, technical, legal, and ethical challenges persist and are discussed. 

## 2. AI in Abdominal Imaging: Pathology Identification and Diagnostic Techniques

The role of AI in diagnosing and characterizing liver, pancreatic, gastric, colorectal, and other abdominal diseases is constantly evolving, bringing new opportunities for early diagnosis, accurate characterization, more precise treatment, and better outcomes. In the following sections, an exploration of the impact of AI on various abdominal pathologies is presented, with a specific focus on hepatic, pancreatic, gastric, and colorectal pathologies. Finally, AI’s advancements to diagnose other abdominal pathologies, including conditions affecting the kidneys, adrenal glands, and spleen, are also discussed. Each section will detail how AI improves diagnostic accuracy, disease management, and patient outcomes across these various areas.

### 2.1. Hepatic Pathologies

**Figure 2:**
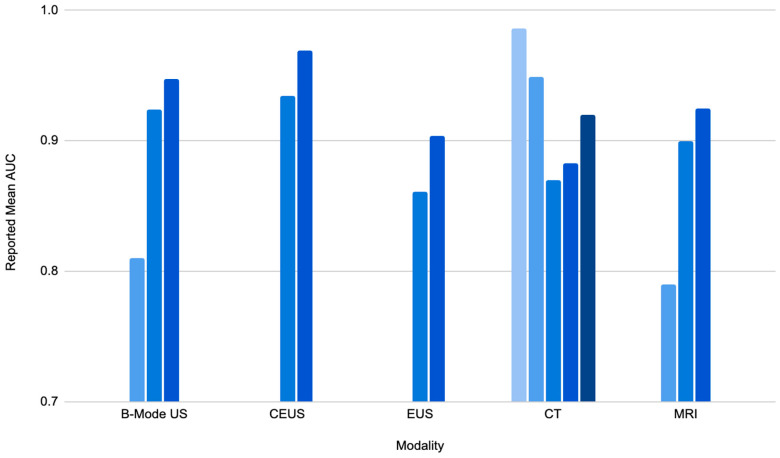
Reported AUC values for AI model diagnostic accuracy of liver lesions by modality.

In the specific context of hepatic pathologies, AI has notably enhanced the identification and description of hepatic lesions, including hepatocellular carcinoma (HCC) [5,6,7,8,9,10,11,12,13,14,15,16,17,18,19,20,21,22,23,24]. HCC is the most common form of liver malignancy, making up 80–90% of all liver cancer diagnoses [5]. Conventional techniques depend on radiologists to differentiate malignant from benign lesions, a challenge due to the heterogeneity of the liver. AI, when trained on large databases of liver images, can pick up on these finer details, leading to better diagnostic results. Training AI models involves exposing the system to many images with known diagnoses, allowing it to learn the patterns and features that distinguish different conditions. For example, in HCC diagnostic performance, area-under-the-curve (AUC) values are commonly reported to stand at over 0.900 across five imaging techniques: B-mode ultrasound (US), contrast-enhanced US, endoscopic US, CT, and MRI [7,8,9,10,11,12,13,14,15,16,17,18,19,20] (Figure 2), with a few exceptions [21,22,23,24]. For example, contrast-enhanced US images read with an AI model achieved an AUC of 0.969, whereas the expert radiologist AUC values for these same images were 0.864 to 0.935 [13]. Furthermore, the accuracy of HCC diagnosis using CT images with AI assistance was greater than radiologists with similar experience who did not have access to the AI tool [17,24]. Overall, these high AUC values suggest that AI can enhance the diagnostic precision of HCC more than conventional approaches (Table 1 and Table 2). 

For values that were reported as a range, the mean value was used. The above data are also reported in Table 1.

Another significant achievement is the use of AI to predict HCC with biomarkers. Models can study imaging data to detect molecular changes and biomarkers linked to HCC and help plan the proper course of action [25,26]. This capability allows for developing individualized and precise treatment plans that help increase the treatment success rate and pave the way for a more practical approach to patient care.

AI algorithms have also shown promising results in accurately predicting HCC progression and the risk of relapse [27,28]. By leveraging these concepts, it becomes feasible to forecast the future course of the disease based on data obtained from imaging techniques, histopathological examination, and molecular markers. This predictive capability is pivotal in guiding follow-up and management plans and determining the timing of interventions, instilling a sense of hope for the future of medical imaging and the potential it holds for improving patient outcomes [28,29]. 

Beyond the applications of AI for HCC, other common liver pathologies, such as liver steatosis [29], fibrosis [30,31,32,33], hepatic ascites [34], and non-alcoholic/metabolic-associated fatty liver disease (NAFLD/MAFLD) [33] have been identified and described using AI-assisted imaging. For example, one study showed that AI-assisted ultrasound can improve accuracy in diagnosing liver steatosis [29]. In addition, AI assistance in liver fibrosis staging using CT images has shown great potential [30,31,32]. AI has demonstrated efficacy in assisting with hepatic ascites’ detection and assessment using CT scans [34,35]. In a systematic review, AI-assisted ultrasound was found to significantly improve the sensitivity and specificity of NAFLD diagnosis [33]. Furthermore, AI has shown the potential to improve the quantification of the liver iron concentration (LIC) through non-invasive imaging, such as the use of MRI rather than the invasive liver biopsy [36] (Table 3). 

The integration of AI in hepatic imaging significantly contributes to the early diagnosis, exact identification, and precise management of diseases, leading to better patient outcomes. By combining the human knowledge and skills of radiologists with the analytical ability of AI, the diagnosis becomes more comprehensive, accurate, and predictive. The result is reassurance surrounding imaging accuracy and the value it adds to managing HCC, liver steatosis, fibrosis, and hepatic ascites.

### 2.2. Pancreatic Pathologies

Pancreatic cancers are quite a challenge due to a majority being diagnosed at a later stage. Pancreatic ductal adenocarcinoma (PDAC) has one of the worst prognoses as a result [37,38,39]. Additionally, pancreatic cancer diagnoses have doubled in the past 25 years and are expected to continue to rise, therefore emphasizing the importance of early detection [37]. AI models have been designed to improve the imaging diagnosis of PDAC using CT scans [40] and endoscopic US [41,42]. Endoscopic US with AI has been found to have a high sensitivity and specificity in the diagnosis of PDAC against other pancreatic masses [41,42,43]. Also, pancreatic cancer detection with artificial intelligence (PANDA) measures high accuracy in detecting pancreatic lesions in non-contrast CT, indicating better results than radiologists regarding sensitivity and specificity [20].

**Figure 3:**
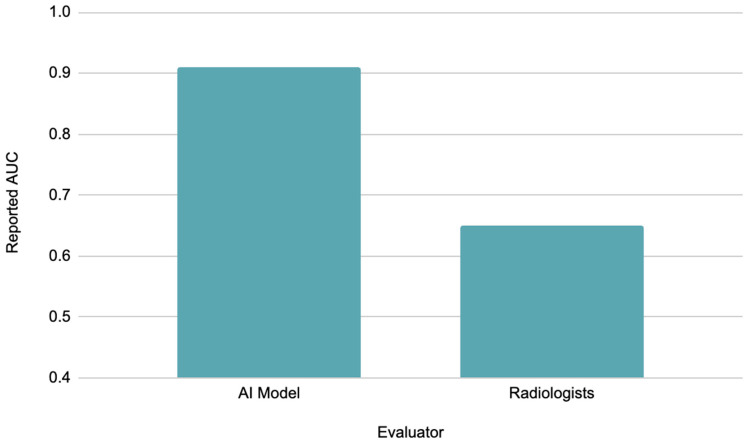
Reported AUC for AI model vs. radiologists for detection of lymph node metastasis from PDAC.

The application of AI is not limited to early detection but also involves prognostic assessments of treatment response and relapse probabilities. AI-based deep learning models have been shown to accurately predict the grading of PDAC tumors using ^18^F-FDG-PET/CT scans obtained before surgery [44]. In addition, these deep learning models can predict prognoses and guide further actions in treatments through analysis of data found in relevant imaging. For instance, a recent study showed that AI could estimate treatment and recurrence probability outcomes and help develop target treatment plans for patients with PDAC [45,46]. Furthermore, AI has been shown to predict PDAC lymph node metastases on CT scans with an AUC of 0.91, whereas radiologists produced an AUC of only 0.65 (*p* < 0.05). This finding suggests that AI can detect lymph node metastasis from PDAC with greater discrimination than radiologists and, therefore, presents a promising tool to be developed and integrated [47] (Figure 3). 

In addition, research has demonstrated the ability of AI to diagnose occult preinvasive cancer that cannot be seen with the naked eye on pre-diagnostic CT images, an important use for early diagnosis. One study noted that an AI model trained on a large dataset could accurately identify pancreatic cancer on diagnostic CT scans and visually occult preinvasive cancer on pre-diagnostic CT scans [40].

Overall, AI has significant potential to assist in diagnosing and differentiating PDAC and occult cancers early, formulating prognostic assessments, predicting tumor grading and treatment responses, and predicting the likelihood of relapse with greater accuracy than traditional methods. By integrating AI into clinical practice, there is potential for more personalized and effective treatment plans, ultimately improving patient outcomes across a range of pancreatic conditions (Table 4). 

### 2.3. Gastric and Colorectal Pathologies

In gastrointestinal imaging, AI improves understanding of the position and size of tumors, inflammatory changes, and other abnormalities. AI has considerable advantages for diagnosing and managing gastric and colorectal cancers by endoscopy. The use of AI in endoscopy—both EGD and colonoscopy—remains highly sensitive and specific in diagnosing colorectal polyps and neoplasms within the GI tract. For instance, AI systems have been found to increase the adenoma detection rate and decrease the adenoma miss rate and quality of endoscopy [48].

In colorectal cancer, AI has been applied to detect biomarkers with prognostic and predictive value from routine images and histopathological slides to help in the decision-making process in the treatment plan [49,50]. The AI models can detect the neoplastic changes in the early stages in endoscopic images, facilitating the timely management of the diseases and improving the patient’s prognosis [51,52]. This is particularly relevant, as colorectal cancer takes the lives of many individuals each year, outnumbered only by the mortality of lung cancer [49,50,51]. 

In addition, AI has been used in endoscopic diagnosis of esophageal and gastric cancers, with evidence demonstrating improved diagnostic accuracy and a decreased miss rate of lesions [53,54,55]. AI has shown the ability to predict histologic features of early gastric tumors, such as the magnitude of differentiation and depth of invasion, using images and videos of specimens [56]. However, AI has shown less efficacy in detecting metastases from gastric adenocarcinoma in a recent study, emphasizing the need to further investigate its utility in this realm [57].

**Figure 4:**
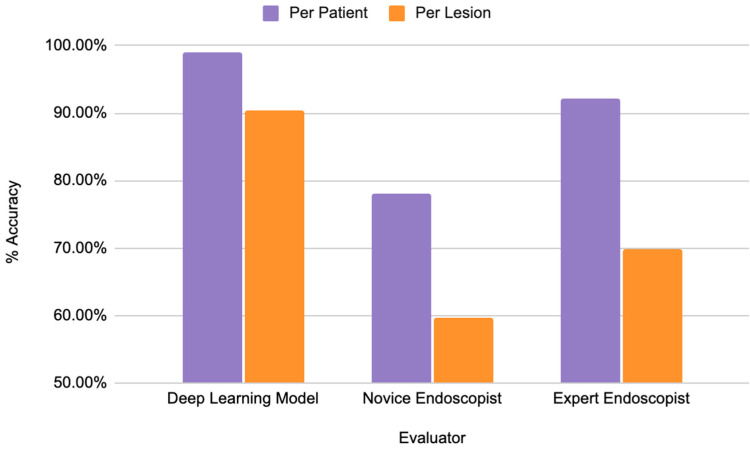
Comparing accuracies of a deep learning model to novice and expert endoscopists for IBD diagnosis.

Beyond the use of AI for cancerous lesions, it has shown efficacy in several other gastric and colonic pathologies. AI has demonstrated great success in identifying the presence of Helicobacter Pylori (*H. pylori*) [58] and diagnosing gastritis using endoscopic images [59]. Furthermore, AI has shown the potential to detect mesenteric and celiac artery bleeding with angiography images [60,61]. Using abdominal images of pediatric patients, AI has been shown to identify necrotizing enterocolitis with similar accuracy to physicians, suggesting a potential application for quickly recognizing this potentially fatal condition [62]. AI algorithms have also shown notable accuracy in detecting intussusception in pediatric patients on abdominal X-rays [63,64] and grayscale ultrasound images [65]. Furthermore, deep learning models have been used to differentiate between Crohn’s disease and ulcerative colitis using endoscopic images, with accuracies and reading times surpassing those of experienced physicians [66,67,68]. Specifically, the accuracy of these deep learning models has been reported as high as 99.1% and as low as 90.4%, while expert endoscopists range from 69.9% to 90.2% and novice endoscopists from 59.7% to 78% [66] (Figure 4). Moreover, small bowel obstructions have been recognized with significant accuracy with machine learning on CT imaging [69,70] and abdominal X-rays [71].

Per patient refers to the AI model’s accuracy when taking into account all endoscopic images for a single patient and making a prediction, whereas per lesion refers to the AI model’s accuracy for predicting the IBD diagnosis based on a single endoscopic lesion. 

In summary, AI has the potential to significantly impact the interpretation of gastric and colorectal imaging in a wide variety of contexts and patient populations. In gastrointestinal imaging, AI may help determine tumor position and size. In addition, AI can help with endoscopic diagnosis and treatment of gastric and colorectal cancers. AI functions extend beyond cancerous lesions, such as diagnoses of multiple gastric and colonic conditions, such as *H. pylori*, ulcerative colitis, Crohn’s disease, gastritis, and vascular bleeding. However, further research is necessary to optimize utility across all areas of gastrointestinal imaging (Table 5). 

### 2.4. Other Abdominal Pathologies

AI has also contributed to describing and diagnosing other abdominal pathologies, such as those of the kidneys, adrenal glands, and spleen. In renal imaging, AI improves the detection of masses, specifically renal cell carcinoma, by semi-automating the delineation and characterization of kidney lesions in cross-sectional imaging [72]. In cases of traumatic injury, deep learning models have shown significant potential to identify renal, hepatic, and splenic injuries using CT images [73]. Furthermore, AI has been studied and found to assist with detecting abdominal hemorrhage using CT images [74] and ultrasound images [75,76].

Furthermore, AI has shown the ability to identify functional adrenal masses and differentiate between the different types of tumors based on the texture features of the CT images, a task usually performed by complex biochemical analysis. AI increased radiologists’ accuracy in identifying these adrenal lesions by greater than 10% [77].

In splenic imaging, AI can detect the spleen’s volume to determine if a patient has splenomegaly [78]. It can also be used with ultrasound imaging to diagnose traumatic injuries to the spleen with greater accuracy than radiologist interpretation alone [73,79]. However, splenic injury detection and characterization with AI may be limited, as a recent study showed more remarkable accuracy and specificity for high-grade injuries when compared with low-grade injuries [80].

## 3. Advanced Image Processing and Analysis

### 3.1. AI-Driven Image Enhancement

AI image enhancement is a process whereby techniques are employed to sharpen images to achieve better resolutions. Thus, it provides the enhanced possibility of recognizing specific subtle changes a radiologist may fail to identify. In recent years, iterative reconstruction algorithms based on deep learning models have been introduced to improve the quality of abdominal CT images with reasonable success compared to the prior iterative reconstruction algorithms [81,82,83,84].

The application of AI algorithms to enhance PET and SPECT images has been proven effective in improving image quality. Given the noisy appearances of these modalities, AI can enhance the image resolution and the amount of quantitative data found. Models reduce the number of radiotracers delivered to the patient and the total scanning time, with the same diagnostic performance level achieved [85]. In PET/CT and PET/MRI, AI can also enhance image reconstruction to decrease the scanning time and radiation dosage. The diagnostic accuracy of MR-based attenuation correction is also improved with AI [86].

### 3.2. Quantitative Imaging

Quantitative imaging using AI has significantly advanced the measurement and analysis of tissue characteristics, with profound implications for personalized medicine and treatment planning. AI-driven quantitative imaging involves extracting detailed imaging features that can characterize tissue properties beyond what is visible to the human eye. Other methods, such as radiomics and deep learning, are critical in this area. Radiomics reuses medical images and quantifies them into high-dimensional quantitative features associated with clinical endpoints, including shape, texture, and intensity. Radiomics has been applied in oncology to capture tumor heterogeneity, prognosis of the treatment response, and risk stratification. For example, radiomics can detect slight variations in textural patterns of the tumor that may be linked with the tumor’s malignancy and probable reaction to the treatment and, therefore, contribute to developing an appropriate treatment plan [87].

Deep learning models, particularly convolutional neural networks (CNNs), further enhance this capability by automatically learning and extracting relevant features from imaging data without human intervention. These models can improve the accuracy of tumor segmentation, classification, and treatment outcome prediction [88,89].

Furthermore, integrating AI enhances disease severity and progression assessment by measuring and analyzing body composition metrics. Algorithms can analyze imaging data from modalities, such as CT, MRI, and US, to quantify body composition parameters, including muscle mass, fat distribution, and bone density, which are critical for assessing various health conditions. For example, a previous study demonstrated the value of AI in extracting body composition measures from routine abdominal CT scans. This study used a fully automated approach to measure fat and muscle masses, validating its clinical discriminatory value. The model showed excellent agreement with manual segmentation and could classify sarcopenia and visceral fat, which are significant predictors of mortality in pancreatic cancer patients [90]. This capability allows for more precise and efficient diagnosis, aiding in early detection and treatment planning.

Moreover, a systematic review and meta-analysis on AI for body composition and sarcopenia evaluation using CT scans found that deep learning models for skeletal muscle segmentation were highly accurate. These models facilitate the automated segmentation of body composition, aiding in diagnosing sarcopenia and other related conditions [91]. This computerized analysis reduces the manual effort required from radiologists, allowing them to focus more on image interpretation and patient care. 

Clinical implications of the advancement of quantitative imaging using AI include the ability to make more precise diagnoses based on tumor characteristics, thereby allowing personalized treatment plans. In addition, clear demarcation of margins and an indication of the area with high metabolic activity can assist in radiation treatments, ensuring maximum radiation therapy is delivered to the tumor without harming other non-target tissues [92].

## 4. Workflow Optimization in Abdominal Imaging

### Automation of Routine Imaging Tasks and Integration with Clinical Workflows

The involvement of AI in radiology has been found to enhance or transform radiological processes and activities, including imaging processes, amounts of work, and radiologist efficiency [93]. Current AI applications, such as ChatGPT, in radiology can assist with scheduling, study prioritization, and report distribution, which can assist in taking some of the burdens off the radiologist and allow them to interpret more images and attend to patients [94]. AI has been implemented in patient scheduling and work list management to prioritize patients who require the medical provider’s attention in the shortest possible time [95].

In clinical practice, AI has demonstrated the ability to reduce interpretation times. For instance, a study on chest CT interpretation showed that AI assistance led to a 22.1% reduction in interpretation times, significantly enhancing radiologist efficiency [96]. Moreover, AI can assist in report communication by automatically generating preliminary reports and highlighting areas of concern for radiologists to review. This quickens the reporting process and enhances the accuracy of diagnoses by reducing the likelihood of human error [97]. AI tools can also be integrated into electronic health record (EHR) systems where radiologists receive real-time decision support, reducing their workload [95]. Besides these administrative positions, other non-interpreter quality enhancement positions have been extended to AI, such as reducing variation in subsequent follow-up advice and improving the quality of radiology reports [95]. These applications assist in managing adherence to clinical guidelines and enhancing service delivery to patients. 

In emergencies, it is possible to use AI to perform quick image analysis and sort cases of differing severity, increasing the rates of returning urgent results and making critical decisions. For instance, a recent paper outlined how AI can help trauma and emergency radiologists quickly and accurately analyze medical images and qualitatively determine disease severity by quantizing the morphological image details. This automated prioritization means that the critical cases receive the attention they need in the shortest time possible [13].

AI-based clinical decision support solutions can assist in recommending the proper imaging tests and interpreting complicated imaging information. This integration can help to optimize the patients’ processes and, therefore, enhance the patients’ experience [6,13]. Moreover, there is an ability to train and constantly improve the software with the help of new data obtained from clinical practice, which makes the results more accurate and reliable.

## 5. AI in Abdominal Interventions

### Guidance in Minimally Invasive Procedures

AI can help guide minimally invasive procedures in the abdomen by enhancing precision, safety, and efficiency. AI algorithms play a crucial role at various stages of these procedures, from preoperative planning to intraoperative guidance and postoperative checks. For example, AI creates detailed 3D models of the patient’s abdominal anatomy from imaging data, improving visualization and aiding surgery scheduling. This reassures doctors and patients, instilling confidence in the procedure’s safety. A recent study highlighted how AI generates high-resolution 3D images from CT or MRI scans to aid surgical planning [98].

AI can also assist in guiding surgery by identifying key body landmarks and essential structures. One study demonstrated how deep learning models can identify safe and risky incision areas and reduce the chance of adverse surgical events [99]. This guidance is crucial to procedural success. Additionally, AI can automate certain parts of the procedure, such as suturing and image interpretation [100].

Furthermore, in the field of interventional radiology, a recent review paper succinctly described the current pre-, intra-, and post-procedural applications of AI [101]. AI can assist with pre-charting tasks, predictions of patient responses to procedural interventions, anatomic visualization, as well as virtual reality education and training [101,102]. Other preoperative applications include radiogenomic assistance in preprocedural diagnoses, prognostication, and outcome predictions [103]. AI has shown the potential to predict survival and response to transarterial chemoembolization in patients with HCC [104,105,106]. Intraoperatively, AI has been used to assist with fusion of images [106], as well as augmented reality eyewear, voice and motion recognition systems, and technology to assess device expenses [107]. Furthermore, intraoperative assistance with the orientation and pathways of tumor ablative probes [108] and applications relating to decreasing and tracking radiation exposure in procedures [109,110] have been described. Moreover, following oncologic therapies, AI can assist with CT scan interpretation of the treatment response and tumor sizing [111,112] and predict survival [112].

## 6. Challenges and Limitations of AI in Abdominal Imaging

### 6.1. Technical Barriers

**Figure 5:**
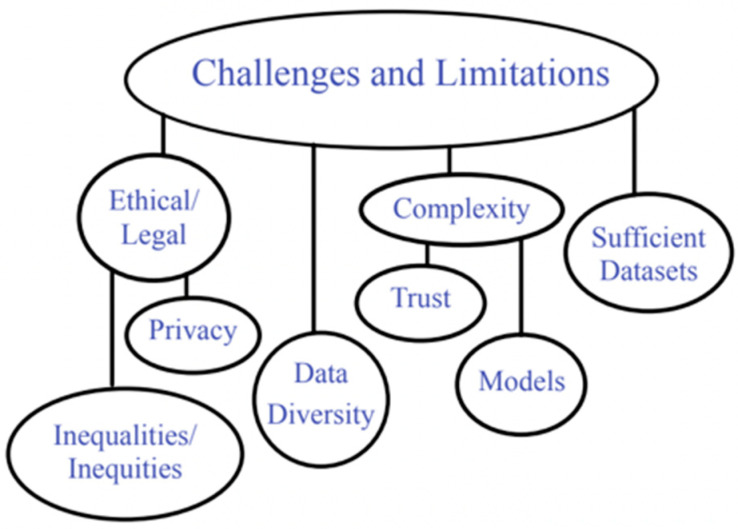
Summary of challenges and limitations of AI in abdominal radiology.

Several technical challenges hinder the integration of AI in abdominal imaging, preventing its widespread adoption and efficacy. A significant obstacle lies in the diversity of imaging data, as variations in imaging protocols, equipment, and patient demographics can impact the performance of AI models. Furthermore, the scarcity of large, labeled datasets for training robust AI algorithms exacerbates this issue [113] (Figure 5). 

Another critical barrier pertains to the protection of patient data privacy. Safeguarding patient information often impedes data sharing, a crucial element in developing and validating AI models. Although researchers are exploring privacy-preserving techniques, such as federated learning, the practical implementation of such methods remains a subject of ongoing investigation [114].

It is essential to consider the complexity of AI models, particularly regarding understanding their decision-making processes. Many AI systems that utilize deep learning operate as “black boxes”, which can pose challenges for physicians seeking to comprehend the rationale behind specific predictions. Establishing trust and promoting the integration of these tools in clinical settings hinges on addressing this need for more transparency. Salahuddin et al. discussed various approaches to enhance deep neural networks’ comprehensibility in medical image analysis. They proposed nine methods for elucidating AI decisions, thereby improving the reliability and applicability of the models [115]. Similarly, Rasheed et al. emphasized the significance of developing comprehensible and trustworthy machine learning models in healthcare. They highlighted the opaque nature of deep learning models as a significant barrier to their adoption in clinical contexts and explored diverse techniques to illuminate the decision-making processes of AI, underscoring their pivotal role in earning the trust of both healthcare providers and patients [116].

An additional technical hurdle arises when AI models demonstrate proficiency with training data but need help adapting to novel information. This challenge often stems from developers utilizing limited or biased datasets during model development. To address this issue, rigorous testing using diverse and representative datasets is crucial [113,114].

In summary, the technical impediments to integrating AI into abdominal imaging encompass heterogeneous data types, privacy concerns, model interpretability, and variations in learning efficacy. Overcoming these challenges is imperative to seamlessly integrate AI into routine clinical practice.

### 6.2. Ethical and Legal Considerations

The use of AI in abdominal imaging raises several ethical and legal considerations that must be addressed to ensure responsible and effective use. Bias and discrimination are two primary ethical considerations. Machine learning algorithms trained on prejudiced datasets may only help replicate healthcare inequalities and produce unequal treatment results. Timely availability of AI technologies is also essential, as there are disparities in the current technologies that need not be perpetuated. Furthermore, privacy and confidentiality are critically important, given the sensitive nature of medical data. Patient data should be handled ethically, and robust systems should be in place to protect privacy as AI technologies are being utilized. Additionally, the clinician–patient relationship may be impacted, necessitating transparency and trust [117,118].

Furthermore, legal liability for AI errors is a significant concern. The ambiguity around who is responsible—whether it is the radiologist, the AI developer, or the healthcare institution—complicates the implementation of AI tools. For instance, Europe has categorized many radiology-related AI tools as high risk, reflecting the potential for significant legal implications [119]. Furthermore, applying product liability law to AI and classifying it as a medical device through regulatory bodies, such as the FDA, adds layers of complexity. Clear rules and oversight are essential in handling these legal issues and ensuring AI tools work well [119,120,121,122].

## 7. Conclusions and Future Directions

The use of AI in abdominal imaging represents a significant advancement in patient care. This narrative review highlighted the role of AI in identifying, diagnosing, and treating abdominal diseases, particularly in organs such as the liver and pancreas. This technology clarifies images, allows for more exact measurements, and augments radiologists’ workflow. These improvements can lead to more accurate diagnoses while also assisting in improving treatment approaches. Lastly, they have the potential to support efficient diagnostic interpretation. Despite the momentum, several challenges persist, including tackling technical limitations, such as differences in data from various training sources and the complex nature of AI systems. Additionally, ethical and legal concerns, such as bias and data privacy, are barriers to seamless AI implementation.

Ongoing research and development should aim to improve AI’s transparency, understanding, and fairness to help a wide range of patients. AI developers and healthcare professionals need to work together to make these improvements when planning and carrying out future clinical trials to assess and boost the use of AI. As technology grows, there becomes a larger and more significant impact on abdominal imaging, leading the way for personalized medicine, better diagnostics, and more effective healthcare. By tackling current challenges and building on progress to date, AI has led to worthwhile changes in abdominal radiology, ushering in a new age of accurate imaging and improved patient care.

## Figures and Tables

**Table 1:** Focal liver lesion classification (benign vs. malignant) on ultrasound images with artificial intelligence assistance compared to radiologists.

Imaging Modality	AI Model AUC	AI Model Sensitivity	AI Model Specificity	AI Model Accuracy	Comparison to Radiologists
B-Mode US [21]	0.77–0.85	80–87%	78%	79–84%	AI model showed greater accuracy than radiologist experts for lesions deemed unknown by the Code Abdomen rating system.
B-Mode US [10]	0.924	86.50%	85.50%	84.70%	Sensitivity and specificity were greater for the AI model compared to experienced radiologists.
B-Mode US [11]	0.947	86.70%	98.70%	82.20%	Not specified
CEUS [12]	0.934	92.70%	85%	91%	AI model displayed greater accuracy than radiology residents and similar accuracy to experienced radiology attendings.
CEUS + Hepatic Markers + AFP [13]	0.969	96.60%	91%	94%	The AUC of the AI model was greater than that of radiologists (0.864–0.935).
Endoscopic US (Image) [14]	0.861	90%	71%	N/a	Not specified
Endoscopic US (Video) [15]	0.904	100%	80%	N/a	Not specified

**Table 2:** HCC diagnosis using CT and MRI images with artificial intelligence assistance compared to radiologists.

Imaging Modality	AI Model AUC	AI Model Sensitivity	AI Model Specificity	AI Model Accuracy	Comparison to Radiologists
MRI [22]	0.79	65–75%	75–79%	73–75%	Sensitivity and specificity of the AI model to identify HCC was similar to that of radiologists, but identification of non-HCC malignancies was inferior to that of radiologists.
MRI [15]	0.9	87%	93%	94%	Less experienced radiologists performed similar to the AI model (AUC 0.893). Expert radiologists outperformed the AI model (AUC 0.957).
MRI [16]	0.925	87.20%	91.60%	N/a	Similar performance of AI model to three experienced radiologists.
CT [17]	0.986	N/a	N/a	83%	Radiologists with access to the AI model had greater accuracy than those who did not have access (79.1% vs. 70.8%).
CT [18]	0.949	N/a	N/a	N/a	Not specified
CT [23]	0.87	75%	88%	61%	AI model had greater accuracy than 2 radiologists (61% vs. 53–55%).
CT [24]	0.883	89%	74%	79.3–81.8%	Radiologists who used the AI model achieved greater accuracy than those who did not have AI assistance.
3-Phase CT [19]	0.92	74%	94%	86%	Not specified
4-Phase CT [19]	0.925	92%	77%	83%	Not specified
CT [20]	0.92	73.9%	96.40%	91.60%	Not specified

**Table 3:** Summary of additional AI capabilities for hepatic pathologies.

Pathology	Findings
HCC Biomarker Prediction	AI models can analyze imaging data to detect molecular changes and biomarkers associated with HCC, enabling individualized treatment planning to increase success rates [25,26].
HCC Progression and Relapse	AI can predict HCC progression and relapse risk based on imaging, histopathology, and molecular markers, aiding in follow-up management and timing of interventions [27,28,29].
Liver Steatosis	AI-assisted ultrasound improves accuracy in diagnosing liver steatosis [29].
Fibrosis Staging	AI applied to CT imaging shows potential in accurately staging liver fibrosis [30,31,32,33].
Hepatic Ascites	AI aids in detecting and assessing hepatic ascites with CT imaging, improving diagnostic accuracy [34].
NAFLD/MAFLD	AI-assisted ultrasound significantly enhances sensitivity and specificity in NAFLD/MAFLD diagnosis [33].
Liver Iron Concentration	AI enables accurate, non-invasive quantification of liver iron concentration via MRI, reducing reliance on biopsies [36].

**Table 4:** Use of AI for pancreatic pathologies.

Application	AI Methodology	Outcome	References
Diagnosis of PDAC	AI with CT, endoscopic ultrasound	High sensitivity and specificity in differentiating PDAC from other pancreatic masses. PANDA model shows higher accuracy than radiologists in non-contrast CT.	[20,37,38,39,40,41,42,43]
Prognostic Assessment	Deep learning with 18F-FDG-PET/CT	Accurate tumor grading, treatment response prediction, and relapse probability outcomes.	[44,45,46]
Lymph Node Metastasis Prediction	AI with CT	Detects PDAC lymph node metastases with AUC of 0.91, significantly higher than the radiologist AUC of 0.65.	[47]
Detection of Occult Preinvasive Cancer	AI on pre-diagnostic CT images	Identifies visually occult preinvasive cancer, aiding in early diagnosis.	[40]
Overall Impact	Multiple modalities	Enhances early detection, prognostic assessments, treatment planning, and relapse prediction in PDAC, facilitating personalized treatment plans for better outcomes.	[37,38,39,40,41,42,43,44,45,46,47]

**Table 5:** Summary of AI applications for gastric and colorectal pathologies.

Application	Outcome	References
Endoscopic Diagnosis	Increases adenoma detection rate, reduces adenoma miss rate, and improves endoscopy quality.	[48]
Detection of Neoplastic Changes	Identifies early-stage neoplastic changes, enabling timely colorectal cancer management using endoscopy.	[49,50,51,52]
Gastric and Esophageal Cancer	Increases diagnostic accuracy and decreases miss rate, and can predict differentiation and depth of invasion of early gastric tumors.	[53,54,55,56]
Detection of *H. pylori* and Gastritis	Accurately identifies the presence of *H. pylori* and diagnosis gastritis using endoscopic images.	[58,59]
Vascular Bleeding Detection	Detects mesenteric and celiac artery bleeding effectively with angiography.	[60,61]
Pediatric Conditions	Diagnosis necrotizing enterocolitis and intussusception in pediatric patients with high accuracy.	[62,63,64,65]
IBD Differentiation	Differentiates Crohn’s disease from ulcerative colitis with high accuracy and reduced reading time with endoscopic images.	[66,67,68]
Small Bowel Obstructions	Recognizes small bowel obstructions accurately, enhancing diagnostic accuracy and supporting quick intervention.	[69,70,71]
Overall Impact	Improves the interpretation of gastric and colorectal imaging across diverse conditions, assisting in cancer and non-cancer diagnoses and treatment. Further research needed in metastasis.	[48,49,50,51,52,53,54,55,56,57,58,59,60,61,62,63,64,65,66,67,68,69,70,71]

## Data Availability

Not applicable.
